# Scar Endometriosis: A Case Report of This Uncommon Entity and Review of the Literature

**DOI:** 10.1155/2013/386783

**Published:** 2013-05-12

**Authors:** Cihangir Uzunçakmak, Ahmet Güldaş, Hasene Özçam, Kemal Dinç

**Affiliations:** Department of Obstetrics and Gynecology, Istanbul Training and Research Hospital, 34098 Istanbul, Turkey

## Abstract

Scar endometriosis is an infrequent type of extrapelvic endometriosis that is rather close together with obstetrical and gynecological surgeries. It is mostly confused with other dermatological or surgical conditions and delays the diagnosis. We report a case of a 50-year-old woman presenting with scar endometriosis 23 years after her last lower segment caesarean section. The epidemiology, diagnosis, pathogenesis, and treatment of the situation are discussed.

## 1. Introduction

Endometriosis is admitted as the presence of endometrial-like stroma and glands outside the uterine endometrial area [1]. It generally occurs in the pelvic sites such as the ovaries, posterior cul-de-sac, uterine ligaments, pelvic peritoneum, bowel, and rectovaginal septum. Extrapelvic endometriosis can be found in unusual places like in the nervous system, thorax, urinary tract, gastrointestinal tract, and in cutaneous tissues unless its most frequent location is the abdominal wall [2]. The main cause of extrapelvic implants is obstetric and gynecological procedures performed during gestation.

There are various theories concerning the scar endometriosis. One of them is the direct implantation of the endometrial tissue in scars during the operation [3]. Under proper hormonal stimulus, these cells may proliferate (cellular transport theory) or the neighborhood tissue may undergo metaplasia, which leads to scar endometriosis (coelomic metaplasia theory). By lymphatic or vascular pathways, the endometrial tissue may reach the surgical scar and then generate to scar endometriosis.

## 2. Case Report

A 50-year-old woman presented in February 2011 with the complaint of pain and swelling on the cesarean scar for one year. Additionally she described cyclic bleeding from this mass for 2 months. She previously had three cesarean deliveries, between 1984 and 1988, and one spontaneous vaginal delivery thirty years ago. She described pain above the cesarean scar that increased during the menstruation period and then noticed a swelling above cesarean scar. She declared mild bleeding from this mass that associated with the first days of her menstruation period.

**Figure 1:**
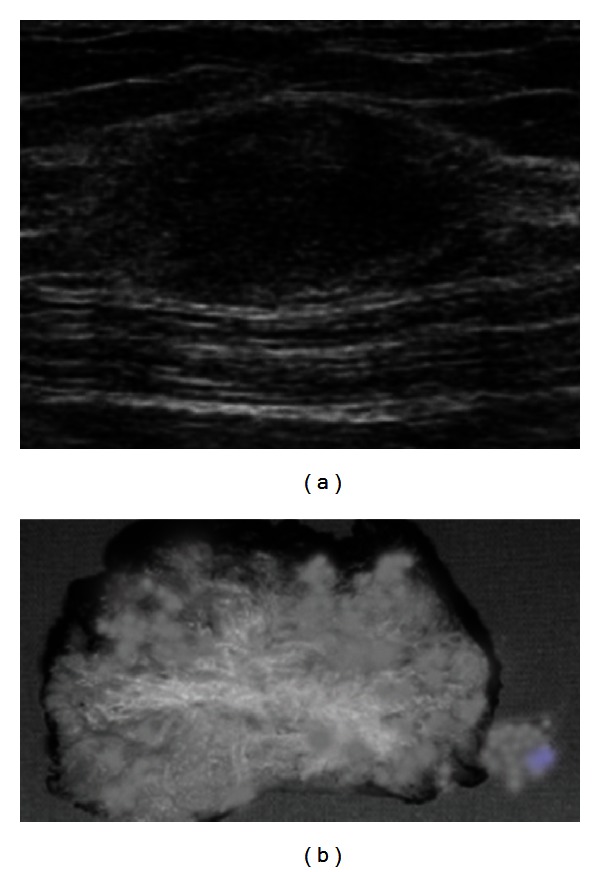
(a) USG in the transverse plane showing echogenic subcutaneous mass. (b) Gross photograph showing grey-white fibrous area with tiny cysts in the subcutaneous fat.

 Examination revealed an approximately 3 cm wide, tender, strict, and immobile right subcutaneous mass beneath the low segment cesarean scar with a little orifice. Transvaginal and transabdominal ultrasound showed a 4 cm × 3 cm × 4 cm, oval-shaped heterogeneous mass within the right rectus abdominus muscle, with no abnormalities of the uterus and ovaries (Figure 1(a)). 

Based on characteristic history and examination findings, behind the most probable choice of endometriosis, other possibilities like hematoma, granuloma, desmoid tumour, and so forth were considered. 

The mass was undertaken through wide excision, and prosthetic mesh was used to close this defect in the rectus sheath (Figure 1(b)). The operation and postoperative consultation were absolute with good functional and cosmetic results.

**Figure 2:**
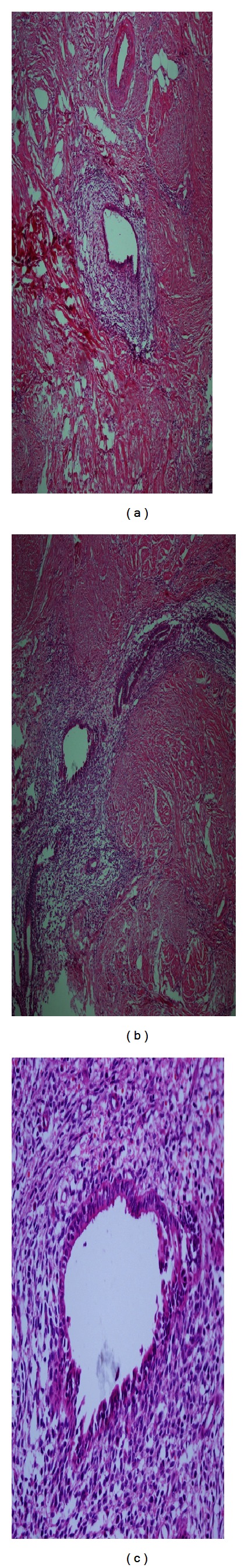
(a) Endometriotic glands and stroma in the subcutaneous tissue. ((b)-(c)) Dense chromatin nuclei with subnuclear vacuoles corresponding to early secretory phase. (Hematoxylineosin strain; original magnification: a, X20; b, X10; c, X40).

Histopathology of the excised mass confirmed the case of scar endometriosis (Figure 2). The patient was examined in the Department of Obstetrics and Gynaecology and not found any recurrence in the first year followup.

## 3. Discussion

Scar endometriosis usually follows previous abdominal surgery, especially early hysterotomy and cesarean section. Minaglia et al. who analyzed 30 years of incisional endometriosis after caesarean section found the incidence of scar endometriosis to be 0.08% [4]. Ectopic pregnancies, salpingostomy puerperal sterilization, laparoscopy, amniocentesis, appendectomy, episiotomy, vaginal hysterectomies, and hernia repair are the other surgical factors for scar endometriosis [5–7]. The reported incidence after midtrimester abortion is about 1% also after cesarean sections ranging from 0.03% to 0.45% [8]. Frequency of scar endometriosis increases by induced number of cesarean section and laparoscopy performed in recent years [9]. 

Direct mechanical implantation seems to be the most plausible theory for explaining scar endometriosis. During caesarean section, endometrial tissue might be seeded into the wound, and under the same hormonal influences these cells proliferates [10]. The endometrial tissue may have certain abilities that make implantation and transplantation during pregnancy. According to this hypothesis, the strongest risk factor for development of scar endometriosis is early hysterectomy like for hysterectomies for abortion [11]. De Oliveira et al. demonstrate that heavy menstrual blood flow and alcohol consumption were positively related to scar endometriosis, and conversely high parity may be a protecting factor [12]. However, direct implantation of endometrial tissue cannot explain all cases. There are few cases of primary cutaneous endometriosis without prior abdominal surgery such as vulva, perineum, groin, umbilicus, and extremities [13], as well as even nasolacrimal localisations [14].

 Clinical diagnosis of scar endometriosis can be made by a careful history and physical examination. The patients present with a mass near the previous surgical scars, accompanied by increasing colicky-like pain during the menstruation [15]. Usually, there is a history of a gynecologic or rarely a nongynecologic abdominal operation. In these patients, correct diagnosis relies on careful examination, right questioning, and obviously taking endometriosis in consideration. 

Furthermore, scar endometriosis is a rare entity, the highlight of this case is the long distant duration from the previous caesarean sections. The interval between the previous caesarean sections and symptoms was 23 years. The patient encountered these worsening symptoms at the perimenopausal age. The underlying reason of this aspect may be the dysfunction of the hypothalamic-pituitary-ovarian axis which becomes a more common finding in peri- and postmenopausal women group. Anovulatory cycles produce no progesterone to stabilize cyclic withdrawal of the estrogen-prepared endometrium, bleeding episodes become irregular, and menorrhagia are common [16]. Also, there are greater risks of benign and malignant neoplastic growths with the increasing age.

 When a proper prediagnosis cannot be achieved, scar endometriosis can be easily mixed with other surgical conditions like hematoma, neuroma, hernia, granuloma, abscess, scar tissue, neoplastic tissue, or even metastatic carcinoma [17], which are a simple excuse to refer the patient to the general surgeon. Often, the diagnosis of endometriosis is not suggested until after histology has been performed. Correct preoperative diagnosis is achieved in 20% to 50% of these patients [18]. 

 The worth of various methods of investigation, such as ultrasonographic examination, computed tomography, magnetic resonance imaging, Doppler sonography, or fine-needle biopsy in the diagnosis of scar endometriomas, is not clear. Imaging procedures help, rather than confirm, in obtaining a differential diagnosis. Ultrasonography is the best and most commonly used investigational procedure for abdominal masses, given its practicality and lower cost. The mass may appear hypoechoic and heterogeneous mass with messy internal echoes. On computed tomography, the endometrioma may appear as a circumscribed solid or mixed mass, enhanced by contrast, and show hemorrhages. Kinkel et al. revealed the sensitivity and specificity of MRI in diagnosing endometriomas to be 90%–92% and 91%–98%, respectively [19]. MRI is also a useful modality for presurgical mapping of deep pelvic endometriosis. Infiltration of abdominal wall and subcutaneous tissues is much better assessed by MRI [20]. Tomographic scans and magnetic resonance imaging are more useful in demonstrating incisional hernias and differential diagnosis [21]. Fine-needle aspiration cytology (FNAC) was reported in some studies for confirming the diagnosis [22]. However, FNAC cytology is a liable method to make the diagnosis of scars, and surgeons must be aware of some diagnosis such as inguinal hernia and reimplantation of potential malignancies during process. Our opinion of FNAC is accurate only in cases of large masses, doubtful diagnosis, and atypical clinical presentations.

 Histology is the hallmark of diagnosis. It is satisfied if endometrial glands, stroma, and hemosiderin pigment are seen [23]. Generally, diagnosis is easy with a microscopic examination of a standard hematoxylin and eosin-stained slide. Furthermore, the cytologist experience must be the important point to clarify diagnosis and to exclude malignancy [24].

 Local wide excision, with at least a 1 cm margin, is accurate treatment choice of scar endometriosis also for recurrent lesions. Recurrence of scar endometriosis seldom happens with only a few cases reported. As expected, the larger and deeper lesions to the muscle or the fascia are more difficult to excise completely. In large lesions, complete excision of the lesion may entail a synthetic mesh placement or tissue transfer for closure after resection [25]. Medical therapy with danazol, progesterone, and GnRH produces only partial recovery, and mostly recurrence occurs after cessation of the treatment with extreme side effects [26]. The incidence of concomitant pelvic endometriosis with scar endometriosis has been reported to be from 14.3% to 26% [27]. Ideally, all patients must be examined for concomitant pelvic endometriosis. At this point, postoperative followup with a gynecologist is preferable.
